# Mechanical behavior of sandstone at various stages under cyclic loading

**DOI:** 10.1038/s41598-025-26994-y

**Published:** 2025-11-07

**Authors:** Zhengyu Sun, Wenjun Meng, Wenhao Dai, Zeping Liu

**Affiliations:** 1 Shanxi Key Laboratory of Intelligent Logistics Equipment, School of Mechanical Engineering, Taiyuan University of Science and Technology, Taiyuan, 030024 China; 2 CNPC Greatwall Drilling Company, Beijing, 100101 China; 3 College of Mining Engineering, Taiyuan University of Technology, Taiyuan, 030024 China; 4 State Key Laboratory of Intelligent Mining Equipment Technology, Taiyuan, 030024 China; 5 Shanxi TZCO Intelligent Mining Equipment Technology Co., LTD, Taiyuan, 030024 China

**Keywords:** Multi-level cyclic loading, Deformation characteristics, Energy evolution, Acoustic emission (AE), Crack, Damage, Engineering, Materials science, Natural hazards, Solid Earth sciences

## Abstract

This study employed a three-level cyclic loading path to investigate the mechanical behavior of sandstone across different failure stages under constant-amplitude cyclic loading. The loading path encompassed the initial crack closure, elastic deformation, and crack propagation stages. The mechanical behavior at each stage was characterized by analyzing deformation response, energy evolution, and acoustic emission (AE) characteristics. The results indicated that the mechanical response of the rock sample exhibited distinct stage-specific characteristics. In the unstable crack propagation stage, the elastic modulus decreased rapidly, the damage energy increased continuously, and both the number of AE events and the associated energy increased significantly. The average peak stress reduction per unit cycle during the unstable crack propagation stage exceeded that during the elastic deformation stage by more than two orders of magnitude. The elastic modulus was highest in the stable crack propagation stage, followed by the elastic deformation stage, and lowest in the initial crack closure stage. Throughout the crack propagation stage, each stress drop was accompanied by a pronounced surge in AE counts and energy, indicating that these stress reductions resulted from the localized propagation and coalescence of internal cracks.

## Introduction

The time-dependent damage evolution of rock under multi-stage cyclic loading is critical to the long-term stability of deep mining operations^1–4^, underground storage seals^5–7^, and slope and tunnel support systems^8,9^. Multi-stage stress paths arising from the coupled effects of engineering disturbances and geologic processes can induce nonlinear hardening, softening, and fatigue damage in rock^10–14^. Such degradation of strength and stability can lead to engineering hazards, including surrounding-rock failure and roof collapse^4,15–17^. Although the multi-stage, high-cycle loading experiments in this study were conducted under laboratory conditions, the damage mechanisms revealed elucidate rock-mass behavior under long-term cyclic loading. In practical scenarios—including deep mining zones, energy-storage reservoirs undergoing cyclic injection–extraction, and slopes subject to seismic or blasting disturbances—rock masses experience high-frequency, multi-stage cyclic stresses^4,16,18^. These stresses lead to progressive damage accumulation, gradual degradation of macroscopic mechanical properties, and time-dependent instability. Therefore, high-cycle fatigue experiments provide valuable engineering insights into the damage evolution of rock masses over their full service life.

Multistage constant-amplitude cyclic loading tests have been established as an effective methodology for investigating the mechanical behavior evolution of rocks under cyclic loading, owing to their ability to simulate progressively varying stress paths. Several systematic studies have contributed to this research direction: Peng et al.^19,20^ conducted five-stage constant-amplitude cyclic loading tests to analyze the influence of parameters such as frequency on the deformation and failure characteristics of sandstone, with particular emphasis on the evolution mechanisms of elastic modulus, Poisson’s ratio, and irreversible deformation across different loading levels; Wang et al.^21^ examined the deformation response and energy evolution of marble with different bedding orientations under multistage cyclic loading, highlighting the role of interstage stress increments in accelerating damage accumulation; Song et al.^22^ compared the mechanical responses of yellow sandstone under 8-stage and 18-stage cyclic loading, integrating stress response, energy absorption, and acoustic emission (AE) characteristics to delineate the mechanical evolution trajectory throughout the loading process; Li et al.^23^ employed AE monitoring to capture signal variations in rock salt during multistage loading and, through multi-parameter analysis—including peak frequency, b-value, and RA-AF distributions—reconstructed the crack propagation and damage evolution processes associated with increasing load levels; Ran et al.^24^ focused on the mechanical response of sandstone under six-stage cyclic loading, providing a systematic analysis of the distinct manifestations of hardening and damage effects across different loading stages. Despite these contributions to understanding the stage-specific mechanical behavior of rocks within the 4 to 18 stage loading range, several limitations remain apparent. First, the number of cycles per loading stage is generally limited to fewer than 50, which is insufficient to capture the fatigue behavior and long-term mechanical evolution of rocks under prolonged cyclic loading. More critically, the selection of load levels in most existing studies lacks a rigorous basis in characteristic stress thresholds—such as crack closure stress, crack initiation stress, and damage stress. This oversight leads to a weak correlation between the external loading path and the internal deformation mechanisms of the rock, thereby hindering the establishment of a coherent relationship between cyclic loading parameters and key deformation stages, including crack closure, elastic deformation, and stable crack propagation. Therefore, the development of a characteristic stress threshold-guided multistage cyclic loading framework is essential to accurately reveal the evolution of mechanical behavior across different damage stages.

Under cyclic loading, the stress and strain in rock samples evolve continuously. Analyzing the stress–strain response during cyclic loading provides a key means of elucidating deformation mechanisms, strength characteristics, and elastic-plastic behavior^24–26^. Fundamentally, this deformation process involves energy transfer and conversion: cyclic loading imparts energy to the rock, with a portion stored as elastic strain energy and the remainder dissipated through irreversible processes such as plastic deformation and crack propagation, thereby manifesting as plastic and damage energies. Therefore, quantitatively assessing the evolution of total, elastic, and dissipated energies during cyclic loading is crucial for elucidating mechanisms of energy conversion and dissipation in rocks^26–28^. Additionally, AE signals that accompany the loading process provide direct insights into internal crack activity^29,30^. Analysis of AE parameters (e.g., events, counts, energy release, amplitude distribution, and b-value) enables non-destructive, real-time monitoring of crack initiation, propagation, and coalescence, thereby enhancing the understanding of micromechanical processes in rocks^30–34^.

This paper presents an investigation of the complete mechanical behavior of red sandstone, from crack closure and elastic deformation to unstable crack propagation, through three-stage cyclic loading tests. It begins by describing the experimental setup and materials, with a particular emphasis on the design of the loading path. It then systematically examines the stress response, modulus evolution, energy conversion, and the AE characteristics of the rock samples.

## Experimental methods and materials

### Experimental procedure

**Step 1**: Six rock samples with the most similar shape, size, density, and P-wave velocity were selected. **Step 2**: Three of these samples were chosen for constant-rate uniaxial compression tests. **Step 3**: The crack closure strain (*ε*_cc_), crack initiation strain (*ε*_ci_), and peak strain (*ε*_p_) were determined from the AE cumulative count-strain curves. **Step 4**: A three-level constant-amplitude cyclic loading path was designed based on these three strain thresholds (*ε*_cc_, *ε*_ci_, and *ε*_p_). **Step 5**: The remaining three rock samples were subjected to this three-level constant-amplitude cyclic loading path. **Step 6**: The data obtained from the tests were analyzed.

### Experimental equipment and materials

**Fig. 1:**
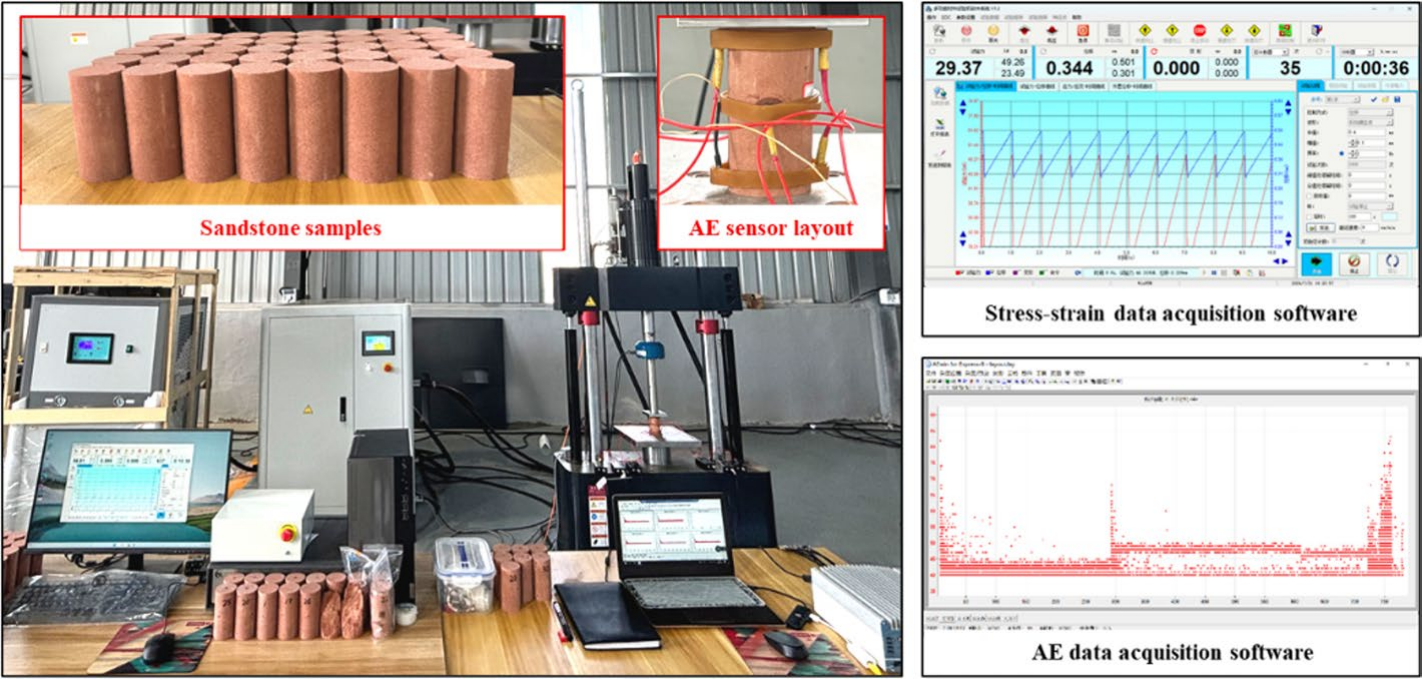
Experimental equipment and materials.

Cyclic loading tests were conducted using a fatigue testing system, which comprised a loading frame, control unit, data acquisition system, and cooling unit. The system featured a maximum axial load capacity of 150 kN and a dynamic loading frequency range of 0–100 Hz. Axial stress and strain were recorded in real-time by built-in sensors. AE signals were captured by six Nano-30 AE sensors^30^. The AE monitoring system parameters were set as follows: a threshold of 40 dB, a pre-amplifier gain of 40 dB, a sampling rate of 1 MHz, and a frequency range of 100–400 kHz (Fig. 1).

The experimental material was fine-grained red sandstone from Neijiang City, Sichuan Province, China, characterized by a uniform texture. To minimize anisotropy, all samples were cored in the same direction from the same fresh, intact rock block free of joints using a dense coring technique. Following International Society for Rock Mechanics (ISRM) recommendations, the samples were machined into cylindrical specimens measuring 50 mm in diameter and 100 mm in height. The end-face parallelism and surface flatness errors were controlled to within 0.05 mm and 0.02 mm, respectively (Fig. 1).

### Experimental design

**Fig. 2:**
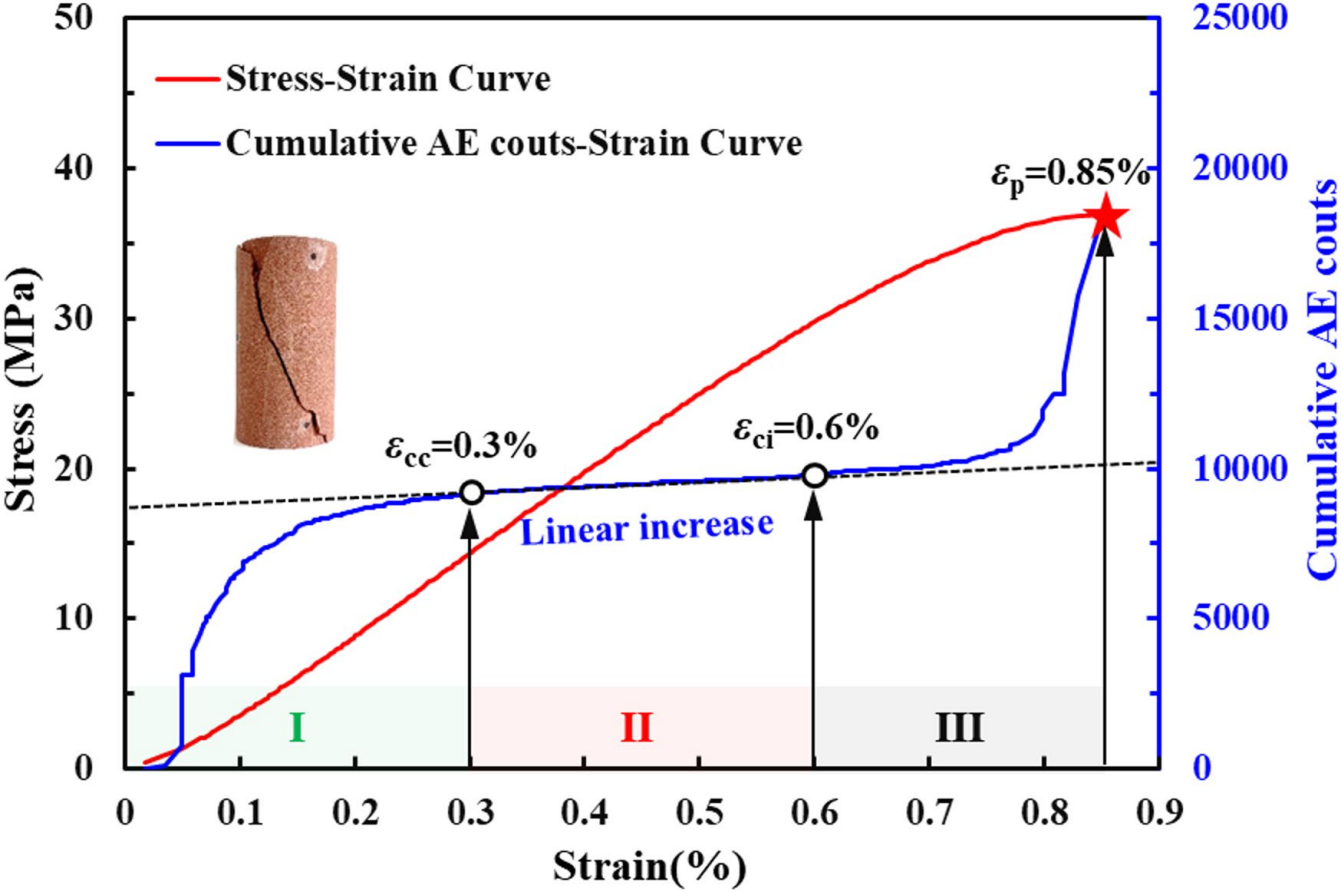
Stress–strain and cumulative AE count-strain curves of sandstone samples under uniaxial compression.

To investigate the mechanical behavior of rocks under constant-amplitude cyclic loading across different damage stages, uniaxial compression tests were first performed to inform the loading path design. The resulting stress-strain and AE cumulative count-strain curves are presented in Fig. 2. Fig. 2 shows that the cumulative AE count evolves through three distinct stages with increasing axial strain: (I) an initial stage of rapid growth, (II) a quasi-linear growth stage, and (III) a final stage of rapid growth preceding the peak stress. This evolution correlates with the development of internal cracks. In Stage I, the rapid increase in AE counts is attributed to the frictional sliding and compaction of pre-existing microcracks, this stage is defined as the initial crack closure stage (ICCS). In Stage II, as most initial cracks are closed and the deformation is primarily elastic, AE activity diminishes, and the cumulative count increases at a nearly constant rate, this stage is defined as the elastic deformation stage (EDS). In Stage III, the nucleation, propagation, and coalescence of microcracks cause a sharp intensification of AE activity, resulting in a steep rise in the cumulative count before failure, this stage is defined as the crack propagation stage (CPS).

The transition points on the AE cumulative count curve from nonlinear to linear growth and from linear to nonlinear growth define *ε*_cc_ and *ε*_ci_), respectively^35,36^. Therefore, the key strain thresholds were determined as *ε*_cc_ = 0.3%, *ε*_ci_ = 0.6%, and *ε*_p_ = 0.85%.

**Fig. 3:**
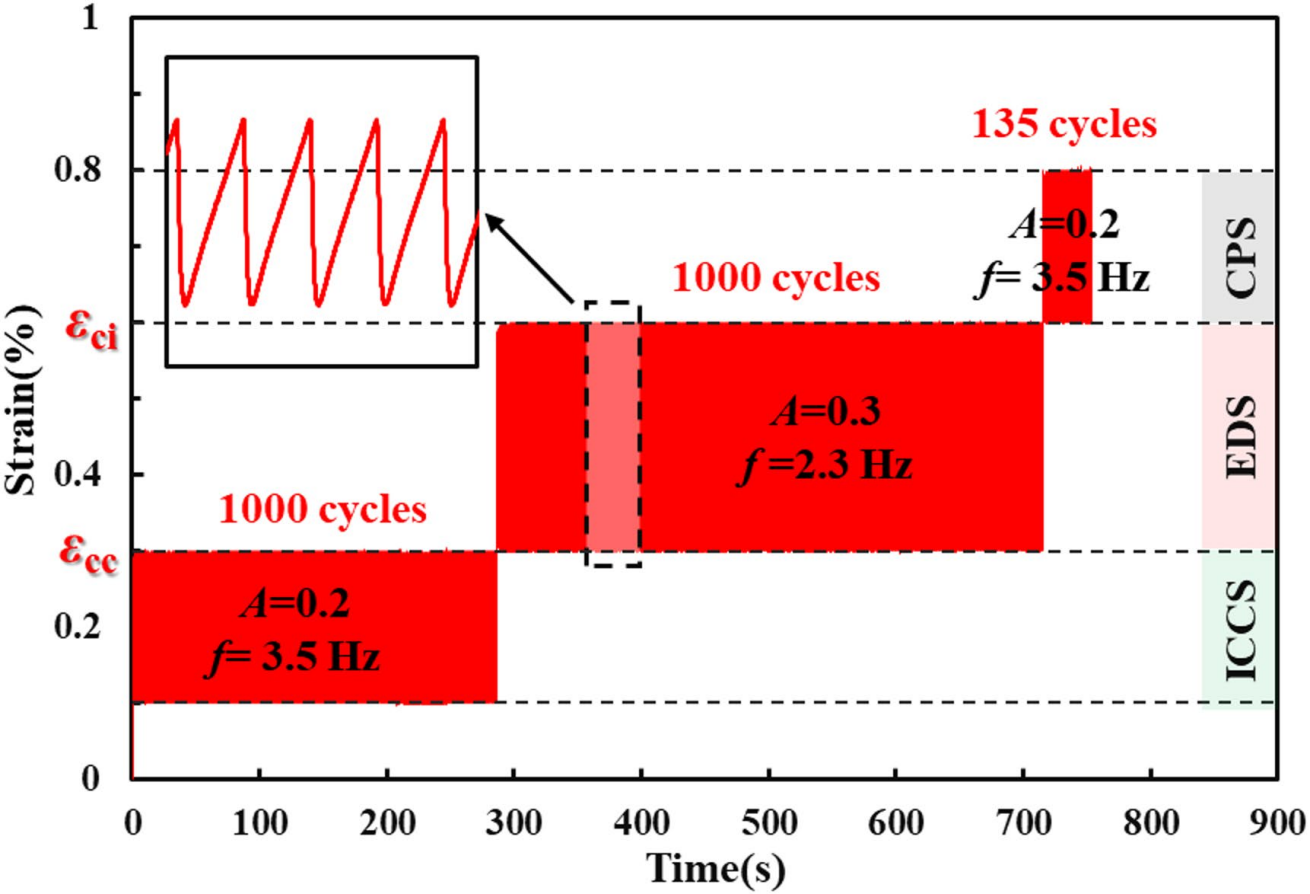
Loading path and waveform used in the experiments.

Based on this stage division and the identified strain thresholds, the three-level cyclic loading path shown in Fig. 3 was designed. The three-level cyclic loads correspond to the initial crack closure stage, elastic deformation stage, and crack propagation stage of the rock sample. The specific loading parameters are listed in Table 1. The cyclic amplitudes for the three levels were 0.2%, 0.3%, and 0.2%, respectively, corresponding to frequencies of 3.5 Hz, 2.333 Hz, and 3.5 Hz at a constant loading rate of 0.7 mm/s. Levels I and II involved 1,000 cycles each, whereas Level III continued until rock sample failure. A sawtooth waveform was used, characterized by a linear loading ramp to the peak stress followed by rapid unloading. All tests were conducted under strain-controlled conditions.

**Table 1:** Experimental loading parameters.

	Parameters			
	Lower limit strain(%)	Upper limit strain(%)	Frequency(Hz)	Number of cycles
Level Ⅰ	0.1	0.3(*ε*_cc)_	3.5	1000
Level Ⅱ	0.3(*ε*_cc)_	0.6(*ε*_ci)_	2.333	1000
Level Ⅲ	0.6(*ε*_ci)_	0.8	3.5	Until damaged

## Results

### Stress-Strain laws

**Fig. 4:**
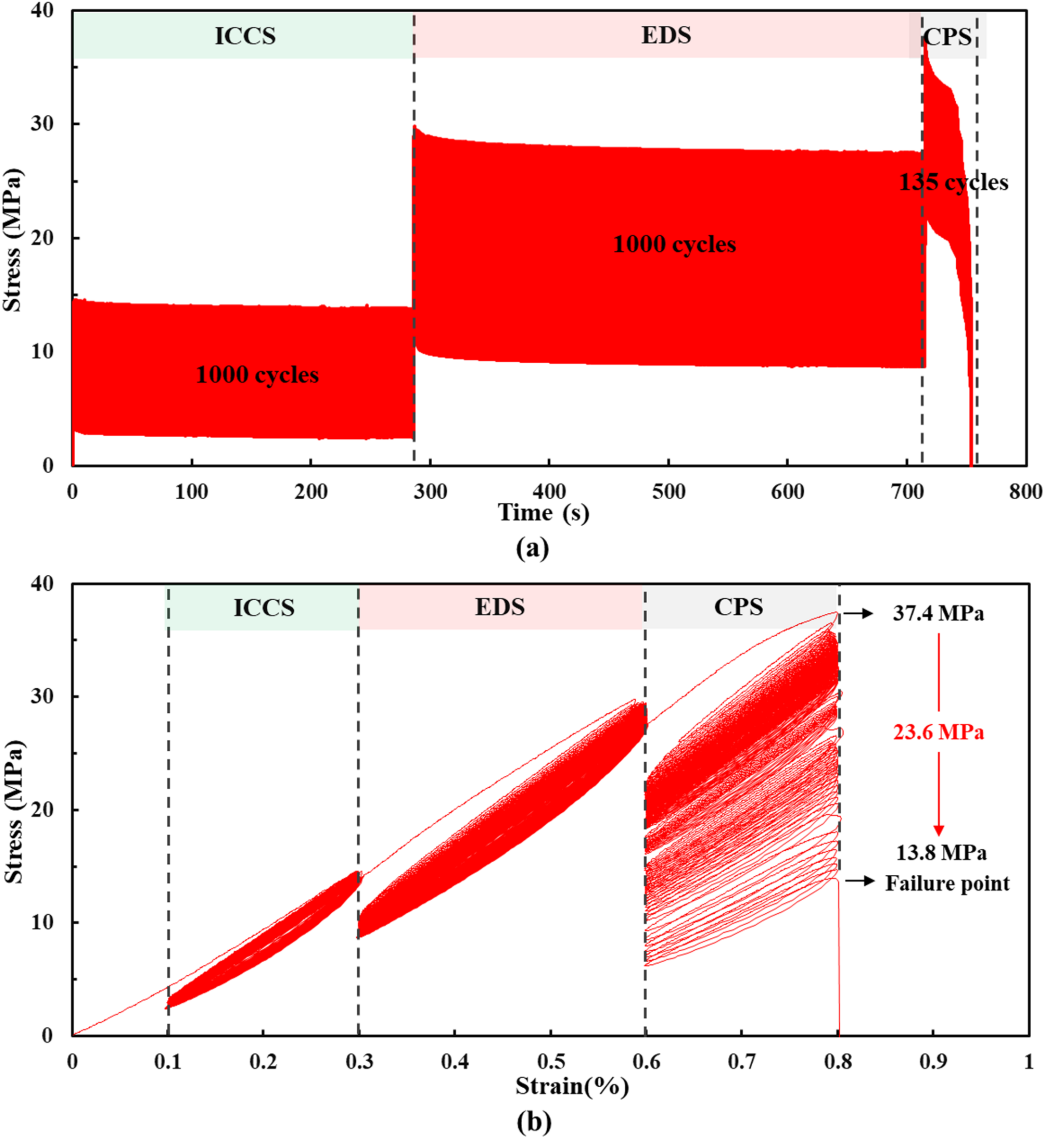
Stress response: (**a**) stress-time curve; (**b**) stress-strain curve.

Stress-strain curves and stress-time curves illustrate the mechanical behavior of rock samples under cyclic loading, such as deformation, yielding, and failure. In this study, the three levels of cyclic loading corresponded to the initial crack closure stage, the elastic deformation stage, and the crack propagation stage of the rock sample, respectively. Fig. 4 illustrates the stress and strain responses of the rock sample at these three stages. During the initial crack closure stage and the elastic deformation stage, the deformation process of the rock sample under 1,000 constant-amplitude cyclic loads remained stable, without a sharp stress decrease. After entering the crack propagation stage, the deformation of the rock sample remained stable during the initial stage of cyclic loading. However, following a sharp stress drop, the deformation process became increasingly unstable, demonstrating progressive instability. During the crack propagation stage, the rock sample failed after only 135 constant-amplitude cycles, as shown in Fig. 4a. Its peak strength dropped sharply from 37.4 MPa to 13.8 MPa, representing a decrease of 23.6 MPa, as shown in Fig. 4b.

**Fig. 5:**
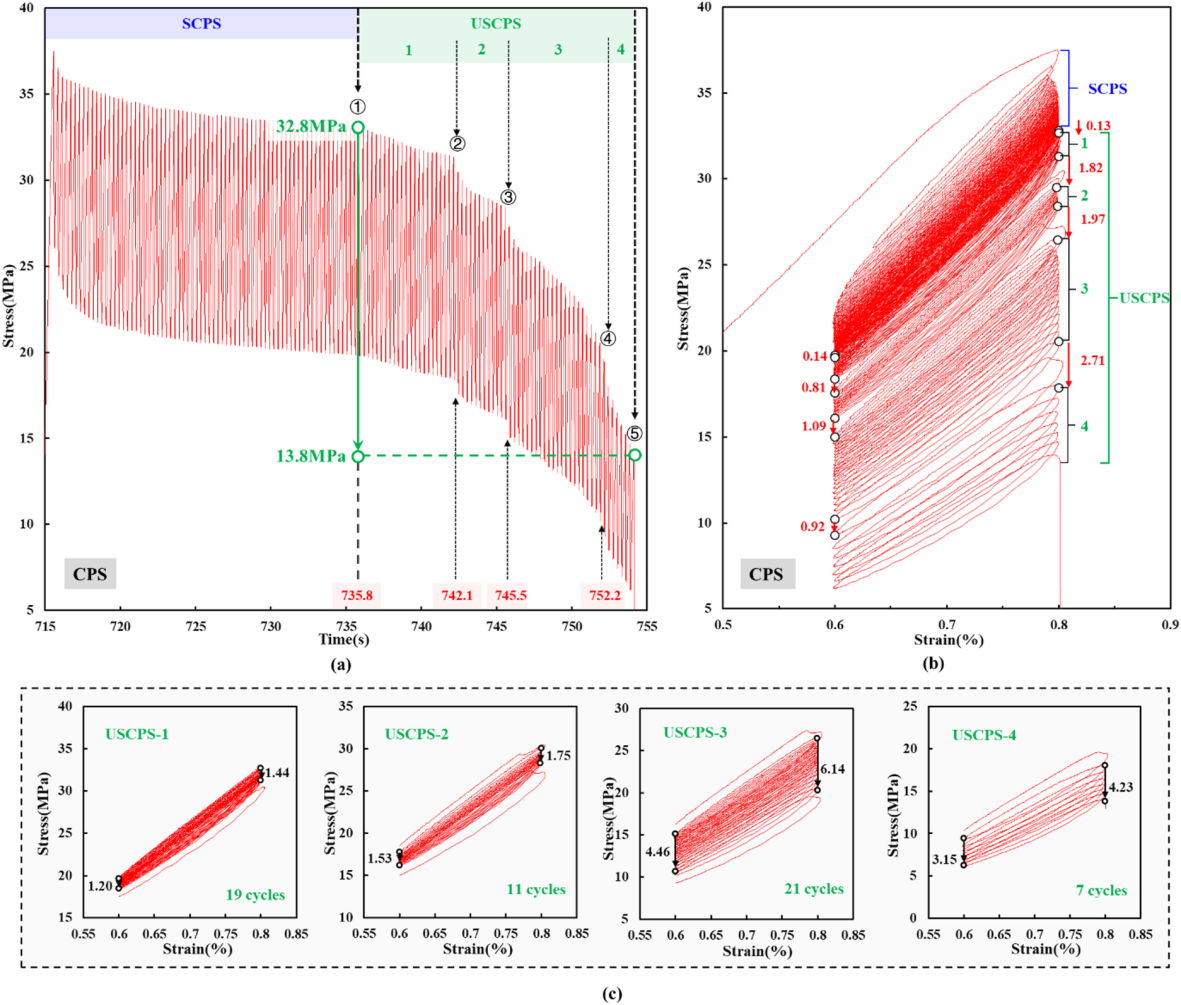
Crack propagation stage: (**a**) stress-time curve; (**b**) stress-strain curve; (**c**) stress-strain curve features and stress drop during the unstable crack propagation stage.

Fig. 5 shows the stress response of rock samples during the crack propagation stage. The deformation of the rock sample remained stable at the beginning of cyclic loading. However, the rock sample underwent an abrupt stress drop after 74 loading cycles. Subsequently, the hysteresis loops became increasingly sparse, indicating progressive instability in the rock sample’s deformation. After four sharp decreases in stress, the rock sample failed, as shown in Fig. 5a. Accordingly, the crack propagation stage was further divided into a stable crack propagation stage and an unstable crack propagation stage^36^, as shown in Fig. 5b. In this study, the stage before the first sharp decrease in stress was defined as the stable crack propagation stage, and the stage after the sharp decrease was defined as the unstable crack propagation stage. Using the sharp reduction in peak stress as a reference, the unstable crack propagation stage was further divided into four segments. The four sharp decreases in stress indicated that the cracks within the rock sample underwent four major episodes of local coalescence and penetration. The increasingly sparse hysteresis loops indicated that the cyclic loading caused progressively more severe damage to the rock sample^37,38^, as shown in Fig. 5c.

**Fig. 6:**
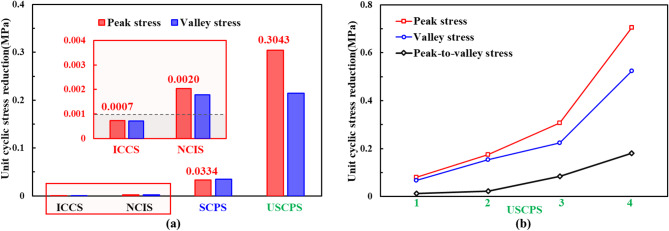
Decrease in stress per cycle: (**a**) decrease at each stage; (**b**) decrease during the crack propagation stage.

Fig. 6a shows the average stress decrease per unit cycle for each stage. During the initial crack closure stage, the stress reduction was minimal, at only 0.0007 MPa, indicating that cyclic loading caused only slight damage. During the elastic deformation stage, the stress reduction was 0.0020 MPa. In contrast, the stress drop was most significant during the crack propagation stage. Specifically, in the unstable propagation phase, the peak stress decreased by up to 0.3043 MPa per cycle, confirming that cyclic loading induced substantial damage during this stage. Fig. 6b shows the progressive increase in the stress decrease per cycle throughout the segments of the unstable propagation stage. This trend indicated not only escalating damage severity within the rock but also that each abrupt stress drop propelled the rock into a more critically damaged state and accelerated the damage rate^39^.

### Elastic modulus

**Fig. 7:**
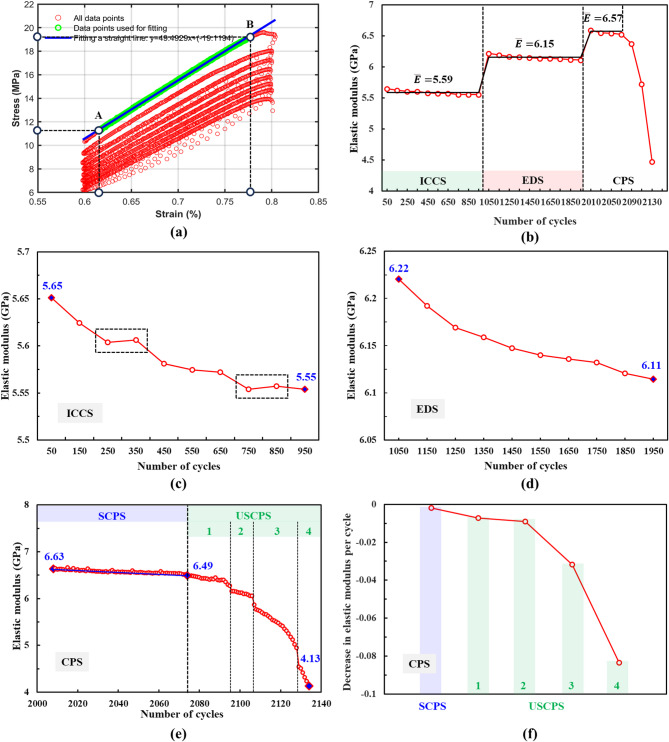
Elastic modulus: (**a**) method for calculating the elastic modulus; (**b**) evolution at different stages; (**c**) evolution during the initial crack-closure stage; (**d**) evolution during the elastic deformation stage; (**e**) evolution during the crack propagation stage; (**f**) average per-cycle decrease during the crack propagation stage.

The elastic modulus characterizes a rock’s ability to resist elastic deformation^40^. A higher value indicates that greater stress is required to generate a unit of elastic strain, reflecting increased stiffness. The elastic modulus was calculated using the following formula^26^:

$$E = (\sigma _{{\text{B}}} - \sigma _{{\text{A}}} )/(\varepsilon _{{\text{B}}} - \varepsilon _{{\text{A}}} )$$

where *E* is the elastic modulus; *σ*_A_ is the stress value corresponding to point A; *σ*_B_ is the stress value corresponding to point B; *ε*_A_ is the strain value corresponding to point A; *ε*_B_ is the strain value corresponding to point B. The calculation principle is illustrated in Fig. 7a.

Fig. 7b shows the variation in elastic modulus across the three stages. The elastic modulus was higher in the elastic deformation stage than in the initial crack closure stage. This indicated that upon entering the elastic deformation stage, most initial cracks and pores within the rock sample had closed or compacted. As a result, the transition in the dominant deformation mechanism, from crack closure to elastic deformation of the rock matrix, enhanced the sample’s resistance to elastic deformation.

Notably, the average elastic modulus during the stable crack propagation stage (6.57 GPa) was greater than that during the elastic deformation stage. However, upon entering the unstable crack propagation stage, the modulus decreased rapidly until the sample failed at 4.13 GPa. In the initial crack closure stage, the elastic modulus exhibited an overall downward trend with multiple rebounds, as shown in Fig. 7c. The elastic deformation stage showed a gradually decelerating decline, as illustrated in Fig. 7d. This was followed by a transition to a linear decrease during the stable crack propagation stage. In the unstable propagation stage, the rate of decline increased significantly, manifesting as four consecutive sharp drops of progressively greater magnitude, as shown in Fig. 7e. Fig. 7f shows the decrease in the unit cycle elastic modulus for each segment of the crack propagation stage. The progressively larger decreases in these values confirmed the acceleration of damage during the unstable crack propagation stage^41^.

### Energy evolution

The experimental equipment repeatedly loaded the rock sample, transferring energy to it until failure. This process demonstrated the conversion and balance of various energy forms, including elastic, plastic, and damage energy. Assuming the experimental system was closed with no heat exchange, the following relationship was derived from the first law of thermodynamics^28,42^:

$$U_{{\text{T}}} = U_{{\text{E}}} + U_{{{\text{DI}}}}$$

where *U*_T_ is the total energy obtained from the rock sample, *U*_E_ is the elastic energy, and *U*_DI_ is the dissipated energy.

**Fig. 8:**
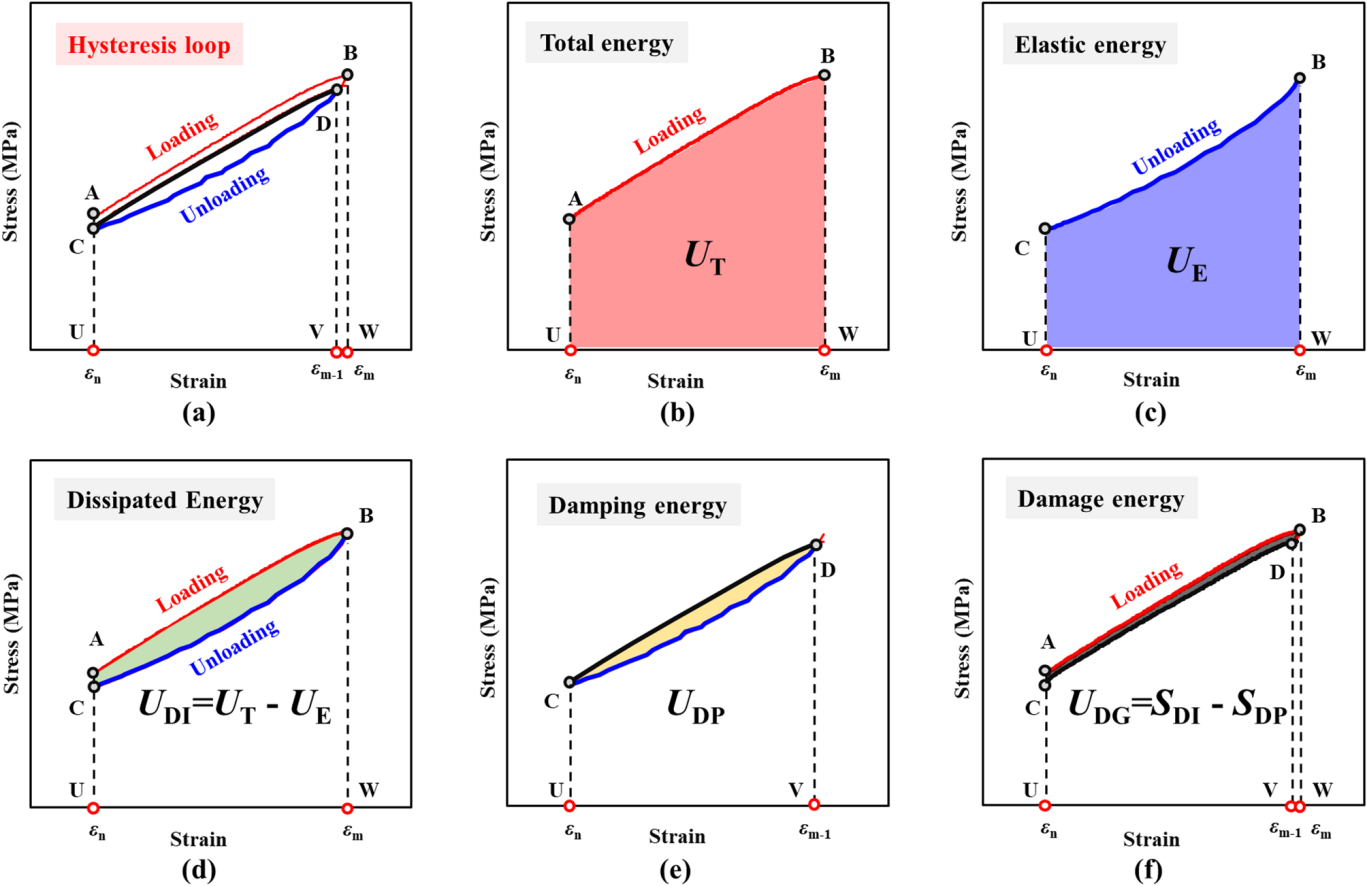
Schematic of energy calculations: (**a**) stress–strain curve; (**b**) total-energy area; (**c**) elastic-energy area; (**d**) dissipated-energy area; (**e**) damping-energy area; (**f**) damage-energy area.

Based on viscoelastic deformation theory, dissipated energy can be further classified into damage energy and damping energy^42,43^. Damage energy is defined as the energy responsible for the initiation and propagation of microcracks, leading to plastic deformation of the rock and a deterioration in its stiffness. Damping energy refers to the strain energy dissipated by overcoming the internal viscous forces within the rock and the friction at the interfaces. The total energy, elastic energy, dissipated energy, damping energy, and damage energy for each cycle were calculated using the stress-strain curve, as shown in Fig. 8.

In a typical cycle depicted in Fig. 8a, loading commenced from point A to point B at a constant rate. During this loading phase, the equipment applied a load to the rock sample, with the area *S*_ABWU_ representing the total input energy *U*_T_, as shown in Fig. 8b. Subsequently, the system was unloaded rapidly from point B to point C, during which the rock sample recovered its elastic deformation. The area *S*_CBWU_ corresponded to the released elastic energy *U*_E_, as shown in Fig. 8c. The difference between area *S*_ABWU_ and *S*_CBWU_, denoted as area *S*_ABCA_, represented the dissipated energy *U*_DI_ for that cycle, as shown in Fig. 8d. Within this dissipated energy, the enclosed area *S*_CD_ was identified as the damping energy *U*_DP_, as shown in Fig. 8e. Consequently, the difference between area *S*_ABCA_ and *S*_CD_ (i.e., area *S*_ABDCA_) equaled the damage energy *U*_DG_, as shown in Fig. 8f. The areas of these irregular shapes were calculated using integration^27^.

$$U_{{\text{T}}} = S_{{\text{ABWU}}} = \int\limits_{{\varepsilon _{n} }}^{{\varepsilon _{m} }} {\sigma _{{\text{L}}} } {\text{d}}\varepsilon$$

$$U_{{\text{E}}} = S_{{\text{CBWU}}} = \int\limits_{{\varepsilon _{n} }}^{{\varepsilon _{m} }} {\sigma _{{\text{U}}} } {\text{d}}\varepsilon$$

$$U_{{\text{DI}}}=S_{{\text{ABCA}}}=U_{{\text{T}}}-U_{{\text{E}}}={\int\:}_{{\epsilon\:}_{n}}^{{\epsilon\:}_{m}}{\sigma\:}_{\text{L}}\text{d}{\upepsilon\:}-{\int\:}_{{\epsilon\:}_{n}}^{{\epsilon\:}_{m}}{\sigma\:}_{\text{U}}\text{d}{\upepsilon\:}$$

$$U_{{\text{DP}}} = S_{{\text{CDC}}} ={\oint }_{{Ln}} \sigma {\text{d}}\varepsilon$$

$$U_{{\text{DG}}}=S_{{\text{ABDCA}}}=U_{{\text{DI}}}-U_{{\text{DP}}}={\int\:}_{{\epsilon\:}_{n}}^{{\epsilon\:}_{m}}{\sigma\:}_{\text{L}}\text{d}{\upepsilon\:}-{\int\:}_{{\epsilon\:}_{n}}^{{\epsilon\:}_{m}}{\sigma\:}_{\text{U}}\text{d}{\upepsilon\:}-{\oint\:}_{L}\sigma\:d\epsilon\:$$

where *U*_DP_ represents damping energy; *U*_DG_ represents damage energy; *σ*_L_ and *σ*_U_ represent loading stress and unloading stress, respectively; *ε*_n_ and *ε*_m_ represent the minimum strain and maximum strain of the nth loading process, respectively; L represents the closed curve formed along the path CDC.

**Fig. 9:**
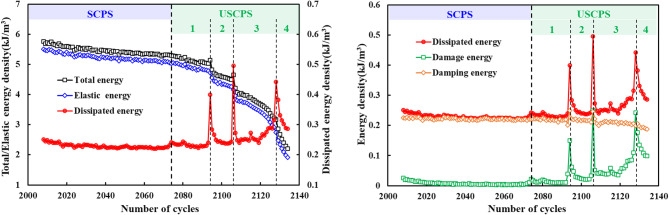
Energy evolution during crack propagation: (**a**) total energy, elastic energy, and dissipated energy; (**b**) dissipated energy, damage energy, and damping energy.

As shown in Fig. 9, the total energy, elastic energy, and dissipated energy varied during the crack propagation stage. During the stable crack propagation stage, the total energy input per cycle decreased linearly, while the dissipated energy remained essentially constant, as shown in Fig. 9a. The damage energy, however, initially exhibited a high value at the start of cycling, gradually decreased, and eventually stabilized. These observations characterize stable crack propagation. However, once the cracks reached a critical extent of stable propagation, the damage energy suddenly fluctuated, indicating the onset of unstable propagation. After entering the unstable crack propagation stage, the total energy input per cycle decreased progressively, and the rate of decrease accelerated. Concurrently, the dissipated energy per cycle began a steady increase, while the damage energy rose markedly, as shown in Fig. 9b.

### AE characteristics

During loading, as the number of cycles or the stress level increases, rock samples exhibit mechanical behaviors such as mineral particle sliding, plastic deformation, and crack initiation, propagation, and coalescence. In this process, the stored strain energy is rapidly released in the form of elastic waves, generating AE phenomena. Thus, analyzing AE parameters, including the number of events, counts, and energy, can provide insights into the damage evolution process in rock samples^30,32,44,45^.

**Fig. 10:**
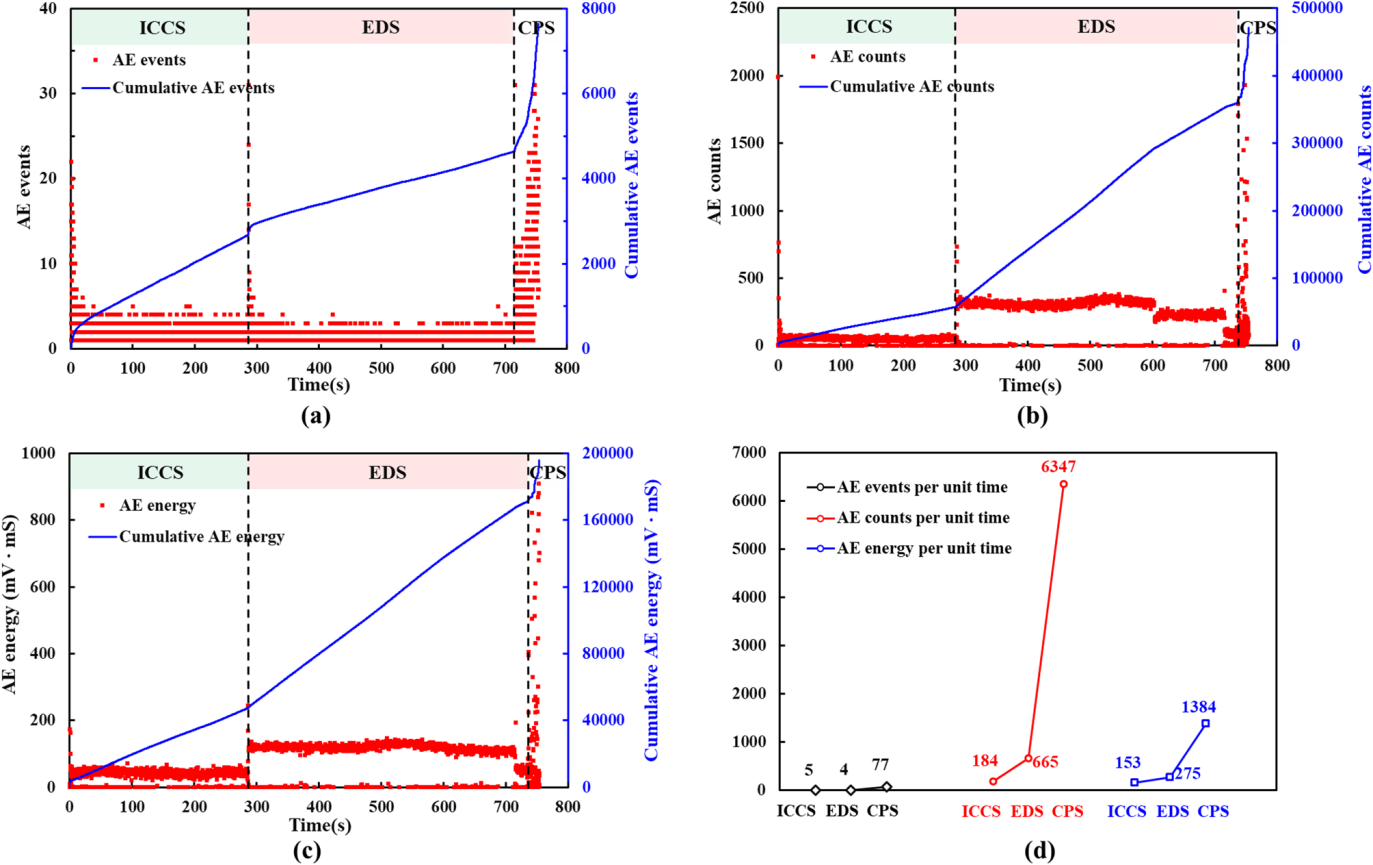
AE events, counts, and energy: (**a**) AE events and cumulative events versus time; (**b**) AE counts and cumulative counts versus time; (**c**) AE energy and cumulative energy versus time; (**d**) AE events, counts, and energy per unit time at each stage.

Fig. 10 shows the changes in AE events, counts, and energy over time for each stage. The growth rates of cumulative counts and energy, along with the count rate and energy release rate per unit time, were higher during the elastic deformation stage than in the initial crack closure stage. In contrast, the growth rate of cumulative events and the event rate per unit time were lower. These observations suggested that during the crack propagation stage, each AE event generated more counts and released more energy. Consequently, during the initial crack closure stage, the rock sample primarily generated friction-type AE events, resulting from repeated friction and closure of pre-existing cracks. Once the crack propagation stage began, the initial cracks had largely closed, leading to a predominance of fracture-type events associated with crack growth.

**Fig. 11:**
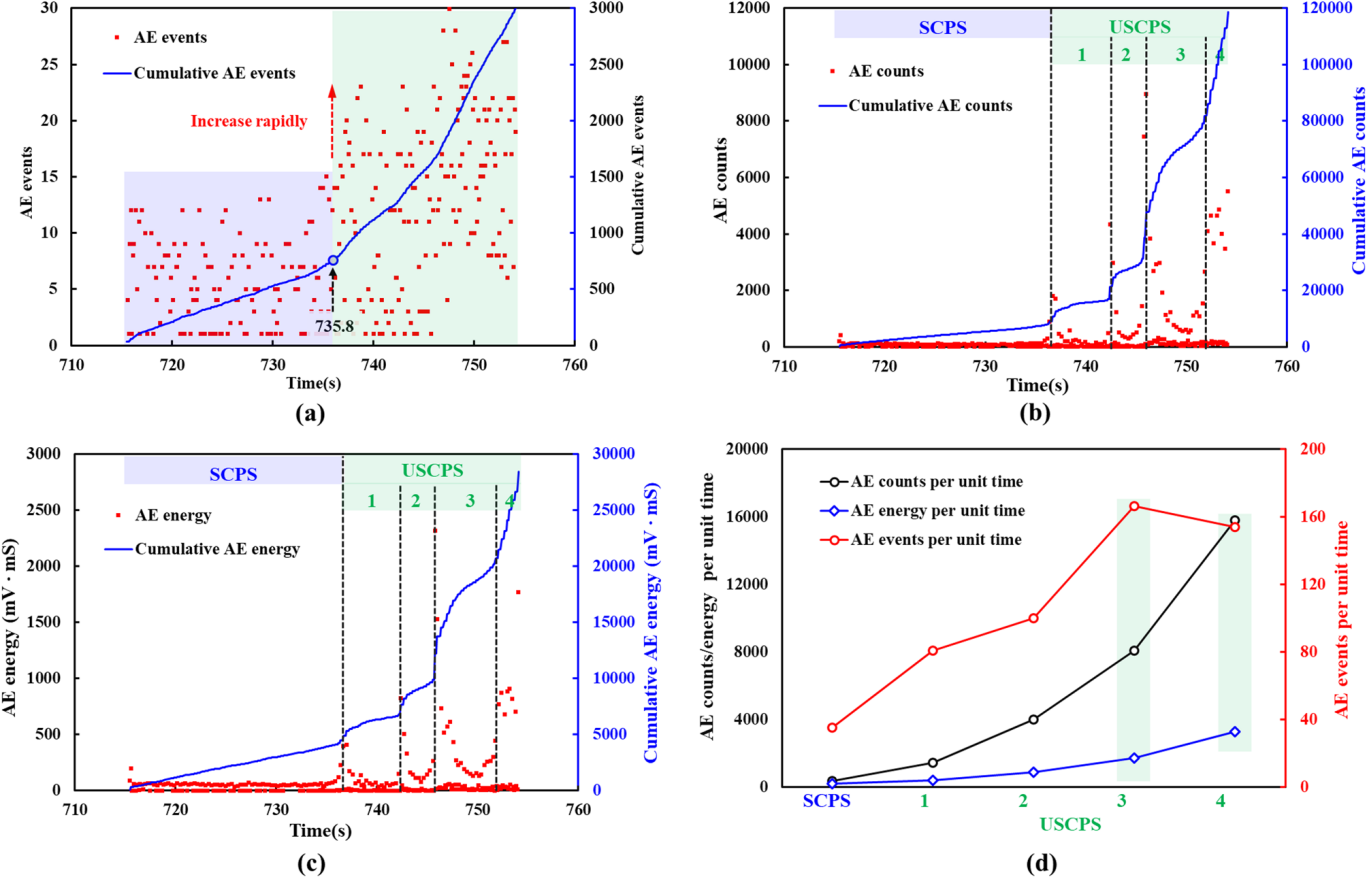
AE events, counts, and energy during crack propagation: (**a**) AE events and cumulative events versus time; (**b**) AE counts and cumulative counts versus time; (**c**) AE energy and cumulative energy versus time; (**d**) AE events, counts, and energy per unit time at each stage.

Fig. 11 presents the variations in AE events, counts, and energy over time during the crack propagation stage. A comparison of the AE cumulative count/energy-time curves with the stress-time curve revealed that surges in cumulative count and energy coincided with stress drops, which confirms the reliability of AE parameters for characterizing internal rock damage. Furthermore, Fig. 11a shows a sharp increase in the event rate after the first major surge, which justified the division of the process into stable and unstable crack propagation stages. As shown in Fig. 11b and c, both cumulative count and energy exhibited linear growth before the first surge, transitioning to non-linear growth thereafter. During the unstable crack propagation stage, the AE count and energy showed three distinct surges, corresponding to three significant increases in the damage energy and notable reductions in the elastic modulus. These observations indicated that three major crack propagation and coalescence events occurred inside the rock sample, intensifying rock damage and leading to substantial reductions in sample stiffness^30^.

It is notable that during the crack propagation stage, changes in the AE event rate did not necessarily correspond to changes in the AE count rate and energy release rate, as shown in Fig. 11d. Compared to USCPS-3, USCPS-4 showed a significant increase in AE count and energy per unit time, while the number of AE events decreased. This suggested a transition in the damage mechanism of the rock sample, from local crack propagation to the formation of a macroscopic fracture surface. During this process, energy release became more sustained, resulting in a reduction in the number of events. Therefore, the number of AE events did not always directly reflect the degree of damage in the rock sample; accordingly, both the count and energy must be considered jointly in damage assessment.

The AE amplitude and b-value are important parameters for characterizing rock damage and crack development^46,47^. AE amplitude refers to the maximum amplitude of the signal waveform, reflecting the magnitude of the source event. The b-value serves as an indicator of internal crack development and the overall extent of rock damage.

In this study, the b-value was calculated based on the influence of the number of cyclic loads and time on rock damage. First, a time step T was set to divide the AE amplitude-time data into consecutive intervals. An amplitude gradient ΔM was then specified, and the frequency N of events within each amplitude range M for every time interval was computed. Finally, a linear regression was applied to the logarithmic relationship between amplitude and frequency, as expressed by the following Eqs. 2^3,26,29^:

$${\text{lg N}}\, = \,a - b \cdot {\text{M}}$$

where *a* is a constant, and *b* is the slope of the fitted curve, which corresponds to the computed b-value. In this study, the time step is set to T = 2 s, the amplitude range is from 40 dB to 90 dB, and the amplitude gradient ΔM is set to 5 dB.

**Fig. 12:**
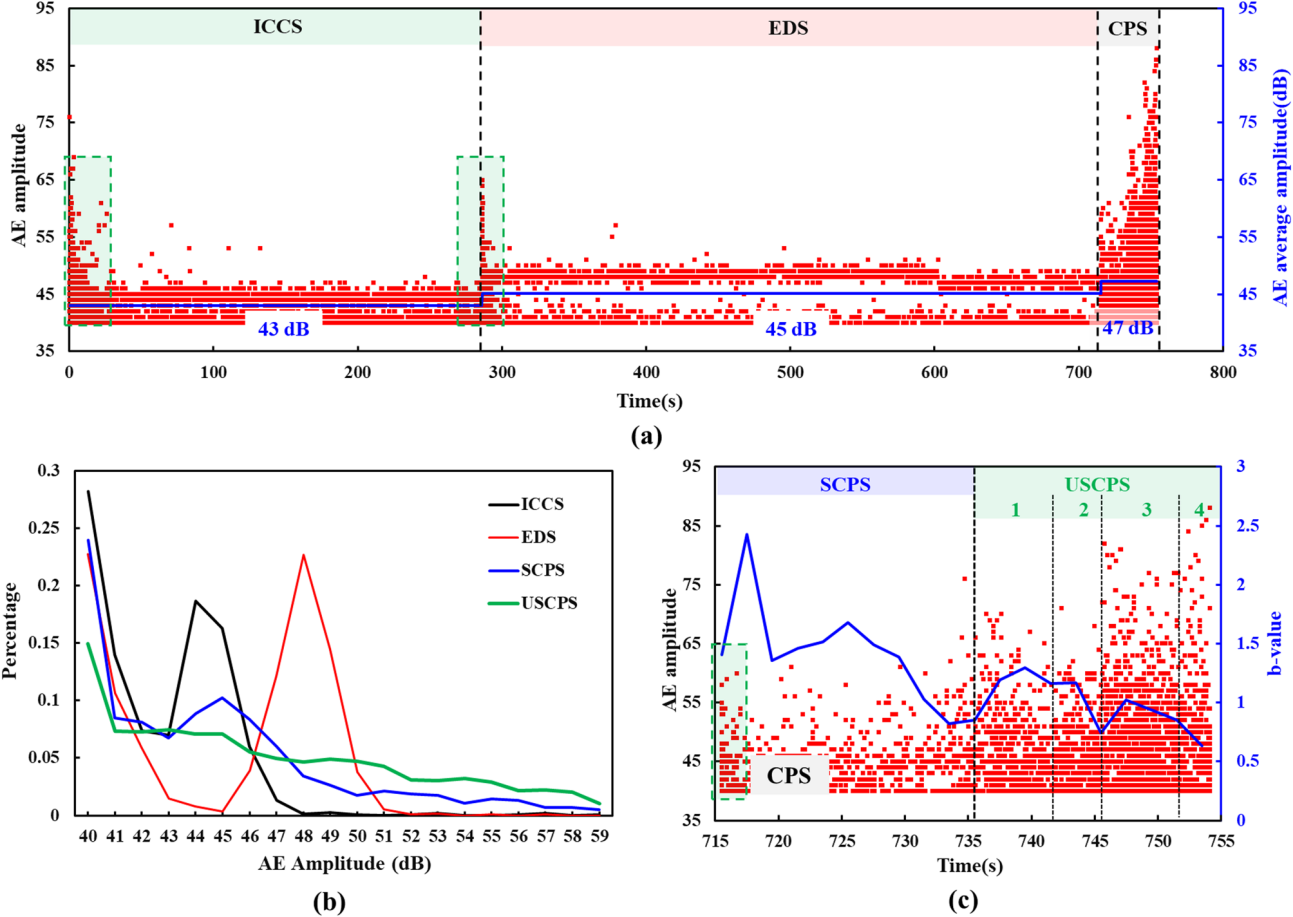
AE amplitude and b-value: (**a**) AE amplitude versus time; (**b**) AE amplitude distribution; (**c**) AE amplitude and b-value versus time during the crack propagation stage.

Fig. 12a shows the variation in AE amplitude with time during loading. The average AE amplitudes were 43 dB, 45 dB, and 47 dB for the initial crack closure, elastic deformation, and crack propagation stages, respectively. The AE amplitude distribution in Fig. 12b indicates that AE event intensity was highest during the unstable crack propagation stage and lowest during the initial crack closure stage. This confirms that the intensity of fracture-type AE events during crack propagation exceeded that of friction-type events generated by the repeated friction and closure of initial cracks. Fig. 12c shows the changes in AE amplitude and b-value over time during the crack propagation stage. The AE b-value displayed an overall decreasing trend, indicating progressively unstable crack development within the rock sample and the eventual formation of large-scale cracks.

## Conclusions

To investigate the staged characteristics of rock deformation and damage, a three-level cyclic loading–unloading path was implemented, corresponding to the initial crack closure, elastic deformation, and crack propagation stages of red sandstone. The use of progressive loading on a single sample effectively minimized the influence of material heterogeneity. The tested red sandstone samples exhibited clearly identifiable crack initiation thresholds and distinct crack propagation stages. The main conclusions are as follows:

1. Upon entering the unstable crack propagation stage, the damage to the rock sample induced by cyclic loading increased substantially. This was manifested as an accelerated decline in elastic modulus, a rapid rise in damage energy, and significant increases in AE parameters.
2. The mechanical response of the sample differed significantly between stages. The average peak stress drop per cycle was 0.3043 MPa during unstable crack propagation, compared to only 0.0020 MPa during the elastic deformation stage—a difference of over two orders of magnitude.
3. Stiffness evolution was clearly stage-dependent. The elastic modulus was highest during stable crack propagation stage, intermediate in the elastic deformation, and lowest during initial crack closure. The closure of pre-existing cracks effectively enhanced the sample’s stiffness.
4. Throughout the crack propagation stage, each stress drop was accompanied by a pronounced surge in AE counts and energy, indicating that these stress reductions resulted from the localized propagation and coalescence of internal cracks.

## Data Availability

The datasets generated during and/or analyzed during the current study are available from the corresponding author on reasonable request. Please kindly contact tyustmwj@tyust.edu.cn to access the data.
